# Polyhydroxybutyrate production in *Bacillus mycoides* DFC1 using response surface optimization for physico-chemical process parameters

**DOI:** 10.1007/s13205-012-0054-8

**Published:** 2012-03-23

**Authors:** Aarthi Narayanan, Karna Venkata Ramana

**Affiliations:** Food Biotechnology Division, Defence Food Research Laboratory, Siddharthanagar, Mysore, 570011 Karnataka India

**Keywords:** *Bacillus mycoides*, Glucose/peptone ratio, Nutrient limitation, Sporulation, Statistical design

## Abstract

The production of polyhydroxybutyrate (PHB) by *Bacillus* sp. is most often growth associated and is influenced by various physico-chemical parameters. Imbalanced nutrient conditions were often found to result in sporulation and low PHB production in *Bacillus* sp. In the present investigation, *Bacillus mycoides* DFC1 strain isolated from garden soil was studied for PHB production in glucose–peptone broth. The effect of glucose/peptone ratio on biomass yield, PHB production and sporulation was investigated. Central composite rotatable design was used to study the interactive effects of three variables: glucose, peptone and pH on cell growth and PHB production. The optimized medium conditions with the constraint ‘to maximize’ cell growth and PHB content were glucose 17.34 g/l, peptone 7.03 g/l at pH 7.3. A maximum dry cell weight of 4.35 g/l and PHB yield of 3.32 g/l amounting to 76.32 % (w/w) of dry cell weight with negligible sporulation at the end of 72 h resulted in a significant increase (1.83–3.32 g/l or 1.82-fold) in the production of PHB in comparison to the medium used in preliminary studies.

## Introduction

Polyhydroxyalkanoates (PHAs) are biodegradable polyesters produced by bacteria which are gaining importance as alternative biopolymers to petroleum-based plastics due to their eco-friendly nature. Bacteria of different genera accumulate PHAs as intracellular carbon and energy storage granules in response to growth media containing excess carbon substrate and limited quantities of nitrogen source (Anderson and Dawes 1990). They are readily degraded by the depolymerases present in the environmental microflora resulting in the formation of water and CO_2_ (Suriyamongkol et al. 2007). Several Gram positive and Gram negative bacteria are widely known to produce PHAs, however, only Gram negative bacteria have been extensively studied. Bacteria such as *Ralstonia eutropha* (*Cupriavidis necator*), *Alcaligenes latus* and other methylotrophs accumulate PHA when cell growth is hampered due to the limitation of nitrogen/phosphorous/magnesium/potassium/oxygen or sulphur in the presence of excess carbon source (Doi 1990). The members of the genus *Bacillus* are reported to accumulate PHB during the growth phase (Borah et al. 2002). PHAs can be subdivided into three broad classes according to the size of monomers. PHAs containing up to C5 monomers are classified as short chain length PHAs (scl-PHA), whereas PHAs containing carbon chain length in the range of C6–C14 and >C14 monomers are classified as medium chain length (mcl-PHA) and long chain length (lcl-PHA) PHAs, respectively (Rehm 2003). In comparison to other PHAs, polyhydroxybutyrate (PHB) is too brittle to be used as plastic material. Members of *Pseudomonas* sp., *Aeromonas hydrophila* as well as *Bacillus* sp. are reported to accumulate heteropolymers of short chain length–medium chain length PHAs (scl-mcl PHA) such as P(3HB-co-3HV), P(3HB-co-3HHx) and P(3HB-co-4HB) when substrates such as γ-butyrolactone or ε-caprolactone are present in the growth medium as carbon source (Labuzeck and Radecka 2001). Unlike the Gram negative bacteria the polymer produced by *Bacillus* sp. are free from endotoxins and can be used for biomedical applications. These tailor-made biosynthesized polymers are considered to exhibit better physical properties and can find use as biodegradable food packaging materials, adhesives as well as biocompatible materials for tissue engineering applications. *B. circulans*, *B. licheniformis, B. amyloliquefaciens*, *B. thuringiensis*, *B. cereus* UW85, *B. megaterium* Y6, *B. sphaericus* ATCC 14577, *B. coagulans* and *B. mycoides* RLJB-017 were reported to produce PHAs in the range 11–69 %w/w of dry cell weight (Chen et al. 1991; Borah et al. 2002; Katricioglu et al. 2003; Yilmaz et al. 2005; Singh et al. 2009). Our experimental studies identified *B. mycoides* DFC1 also to accumulate poly (3HB-co-3HV) copolymer with high hydroxyvalerate (-HV) content of 31.3 mol % (unpublished results). Although PHB was first identified in *Bacillus sp*, until now the large-scale PHB production using *Bacillus* sp. has been met with limited success (Wu et al. 2001; Valappil et al. 2007). *Bacillus* sp. strains endowed with rapid growth and ability to utilize a variety of readily available cost-effective substrates is neglected due to the sporulation interfering with PHB production (Wu et al. 2001). Few studies have documented the accumulation of PHB in *Bacillus* sp. during the late log phase or early stationary phase; however, the accumulated PHB was found to be degraded at the onset of sporulation (Chen et al. 1991; McCool et al. 1996). An optimized medium might enhance and maintain PHA content even at the instance of sporulation in *Bacillus* sp. The parameters such as the carbon (C) and nitrogen (N) source as well as pH of the medium exert an influence on the metabolism of these bacteria and accumulation of PHB (Wu et al. 2001). Response surface methodology (RSM) is an efficient tool used to study the interactive effect of parameters involved in fermentation process seeking optimized conditions for improved product yields (Montgomery 2005; Pal et al. 2010). In the present study *Bacillus mycoides* DFC1, a growth-associated PHB-producing bacteria was grown under conditions favoring PHB production and was investigated for sporulation. Initial pH of the medium and the glucose/peptone (*G*/*P*) ratio was found to support the stable PHB production without reduction in PHB biosynthesis. Few reports on the optimization of PHB production from *Bacillus* sp. has been documented (Valappil et al. 2007; Pandian et al. 2010); however, detailed studies on the correlation of the medium composition and its pH favoring PHB production over sporulation are lacking. The aim of the present study is to optimize PHB production and control the sporulation in *B. mycoides* DFC1 for the enhanced PHB production through statistical media optimization for its possible application in scale-up studies.

## Materials and methods

### Microorganism

The PHB-producing bacterium used in the present study was isolated from garden soil at Mysore, India. The strain was identified by sequencing the 1.4 kb of 16S rRNA gene amplified using universal forward primer: 5′ AGAGTTTGATCCTGGCTAG 3′ and reverse primer: 5′ AAGGAGGTGATCCAGCC 3′. The 16S rRNA gene sequence analysis was carried out using NCBI-BLAST homology search (National centre for Biotechnology Information http://www.ncbi.nml.nih.gov) program and identified the bacterium as *Bacillus mycoides* DFC1. The nucleotide sequence have been deposited with NCBI database under the accession number GQ344802 (Aarthi and Ramana 2011). The glycerol stock culture maintained at 4 °C was used for inoculum preparation.

### Comparison of PHB production in different media

Three different media were investigated to determine the suitable composition supporting maximum PHB accumulation by *B. mycoides* DFC1. The media reported earlier by Ramsay et al. (1990) and Tajima et al. (2003), respectively, were compared with modified glucose–peptone broth whose composition comprise/l: 10.0 g glucose, 5.0 g peptone and 5.0 g sodium chloride for PHB production. Three percent (v/v) of the 18 h culture was used as inoculum in all experiments. The flasks were incubated at 37 °C at 140 rpm for 48 h. The sporulation in the fermentation media at the end of 48 h was determined as the number of heat-stable cells present after heat treatment at 80 °C for 20 min according to Stevenson and Segner (2001).

### Effect of culture conditions on PHB production

Based on the results from preliminary experiments, the glucose–peptone broth (GPB) was used further to identify the appropriate glucose/peptone (*G*/*P*) ratio for PHB production. The initial concentration of glucose was varied from 5.0 to 20.0 g/l and the *G*/*P* ratio of the medium was further adjusted to 1.5–15.0 by varying the concentration of peptone for 15.0 g/l of glucose. The residual glucose was estimated by 3,5-dinitrosalicylic acid method (Somogyi 1952). Based on the preliminary ‘one-factor-at-a-time’ optimization method, the medium containing 3:1 (*G*/*P*) ratio with an initial pH 7.3 resulted in a maximum PHB production. This data was further used to fit in the design of experiments for response surface optimization.

### Experimental design and optimization

The optimization of process parameters in growth-associated PHB production by *B. mycoides* DFC1 was studied using central composite rotatable design (CCRD) of RSM (Stat-Ease, Inc Design Expert software, trial version, 8.03, Minneapolis, USA). The CCRD for the three independent variables: glucose (*A*, g/l), peptone (*B*, g/l) and pH (*C*) each at five levels including 6 replicates at the center point, 6 axial points (*α* = ±1.682) and 8 factor points leading to a total number of 20 experiments was employed for optimization. Each variable was studied at two different levels (−1, +1) and center point (0) which is the midpoint of each factor range. Table 3 shows the minimum and maximum range of variables investigated and the full experimental plan with respect to their actual and coded values. The experimental results were fitted with a second-order polynomial function:1where, *Y* is the predicted response, *b*_0_ the intercept, *b*_1_, *b*_2_, *b*_3_ the linear coefficient, *b*_11_, *b*_22_, *b*_33_ the squared coefficient and *b*_12_, *b*_13_, *b*_23_ the interaction coefficient.

### Data analysis

Design Expert 8.03 (Stat-Ease, Inc, Minneapolis, USA) was used for the data analysis. The response surface model graphs were used to identify the effects of linear, quadratic and interactive terms of the independent variables on the chosen dependent variables. To validate the model, the average of each response (cell dry weight and PHB content) was determined from the completely optimized medium composition in duplicates. The statistical significance of the model was checked by Fischer’s *F* test and the level of significance was given as *p* value.

### Extraction and characterization of PHB

The biomass was separated by centrifugation at 9,000 × *g* for 10 min and washed twice with double distilled water. The biomass was kept in −20 °C overnight and later freeze dried under vacuum for 5 h using Heto Dry winner model DW3 lyophilizer. The cell dry weight (cdw) measured in grams per liter (g/l) of culture broth was determined from the lyophilized biomass. The PHB extraction was carried out by dissolving dried biomass preparations in equal volume of sodium hypochlorite (pH 12.0) and chloroform and incubating the mixture at 37 °C for 1 h. The mixture was filtered and the PHB content defined as the ratio of PHB concentration to cell concentration was quantified from the chloroform phase by gravimetric method (Hahn et al. 1995). The residual biomass or non-PHB content was determined as the difference between the cell concentration and PHB. The polymer samples were analyzed for its purity using Thermo-Nicolet FT-IR spectrometer, Model 5700, (Madison WI). The spectra were recorded in the MID IR range from 4,000 to 650 cm^–1^, using single bounce attenuated total reflectance (ATR) accessory with zinc selenide crystal. Sixty-four scans were averaged to get the spectra. The IR spectra were recorded with 4 cm^–1^ resolution and analysis of the spectra was carried out using OMNIC software.

## Results and discussion

### Identification of a suitable media for PHB production

**Fig. 1:**
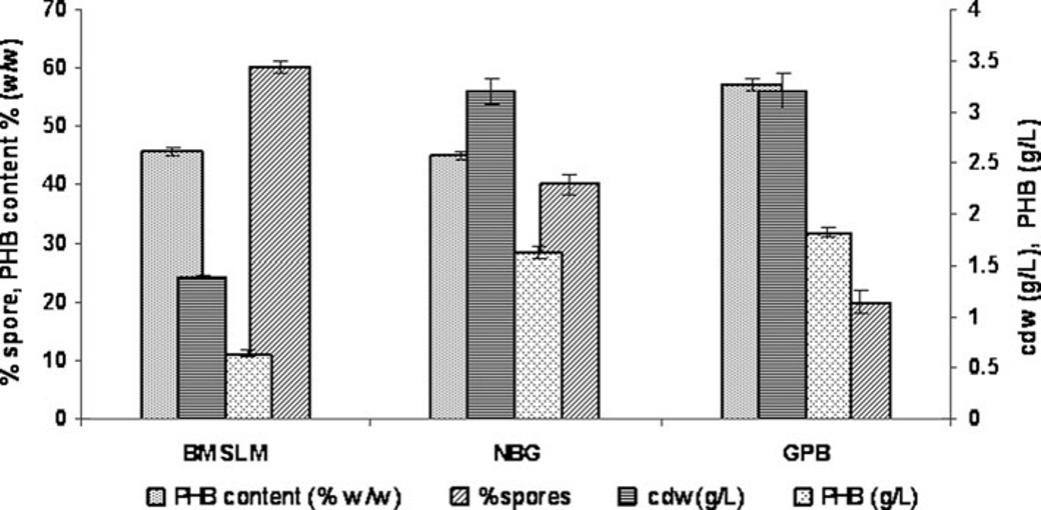
Comparison of cell growth, sporulation and PHB production by *B. mycoides* DFC1 in different media

The yields of biomass and PHB as well as the percentage of sporulation in three different media are shown in Fig. 1. *B. mycoides* DFC1 showed less growth and PHB production in Basal mineral salt medium (BMSLM). The endospore staining revealed >60 % sporulation under nutrient-limited conditions when compared with the other two media. Chen et al. (1991) earlier reported on the degradation of the PHB polymer due to sporulation while Fujita et al. (2005) discussed on the regulation of the gene SpoOA responsible for sporulation during nutrient limitation. The present data also corroborated the interference of PHB production by sporulation in *B. mycoides* DFC1 due to nutrient limitation in BMSLM. Further, the PHB production in GPB medium (1.83 g/l) was equally effective as nutrient broth medium with 1.0 % glucose (NBG medium) of Tajima et al. (2003), (1.62 g/l), indicating that a high PHB accumulation (57.2 %w/w) in the isolated strain, *B. mycoides* DFC1 could be achieved with these media ingredients.

### Determination of initial glucose concentration

**Fig. 2:**
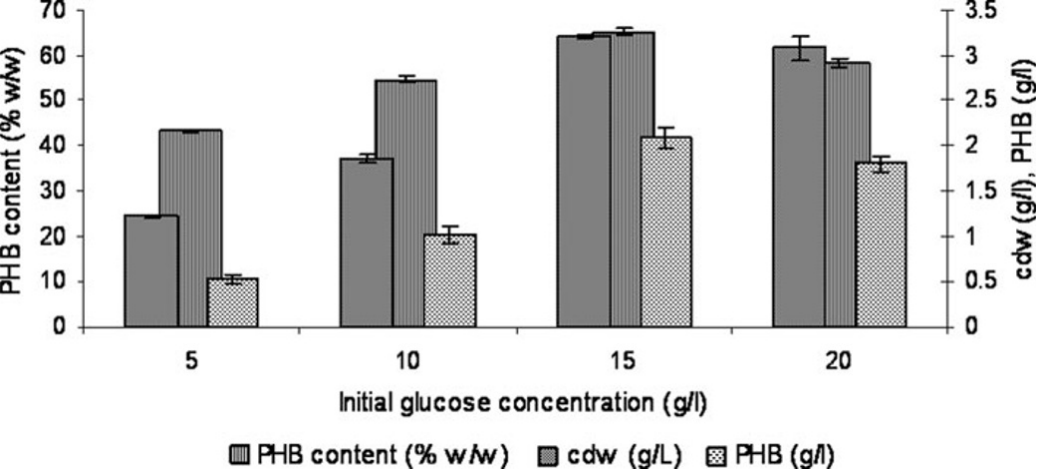
Effect of initial glucose concentration on PHB production in GPB media

The concentration of the glucose in the culture medium determines the cell growth and PHB production. The results for the initial glucose concentration of the GPB medium supporting maximum PHB production is shown in Fig. 2. It is evident that both cell growth and PHB content improved as the initial concentration of glucose was increased from 5.0 to 20.0 g/l. Maximum cell growth and glucose utilization occurred up to 15.0 g/l, as a result the medium became acidic (pH 5.2). Fluorescence microscopic study after every 12 h revealed that the low pH of the medium did not affect the PHB accumulation and resulted in 65.22 %w/w of dry cell weight at the end of 72 h.

### Effect of *G*/*P* ratio and initial pH

In the present study the *G*/*P* ratio was identified to affect the growth-associated PHB production as well as sporulation to a certain extent in *B. mycoides* DFC1. Table 1 show that the 3:1 *G*/*P* ratio supported maximum biomass and PHB content. Studies on the use of protein hydrolysates and other organic nitrogen sources to support good biomass and PHB content has been reported for bacteria such as *R. eutropha*, *Azotobacter beijerinckii* and recombinant *E. coli* (Lee and Chang 1994; Bormann et al. 1998a, b). The extremes of *G*/*P* ratio, i.e. the presence of excess peptone or glucose does not favor for a high PHB content. The use of C/N ratio as an important parameter in fermentation process to achieve high cell density and product formation was documented by Grothe et al. (1999). Similarly, a high C/N ratio favoring maximum PHB accumulation in *Alcaligenes eutrophus* has been reported by Park et al. (1997).

**Table 1:** Effect of *G*/*P* ratio on PHB production

Parameters	*G*/*P* ratio			
	15:1	5:1	3:1	1.5
Dry cell weight (g/l)	0.76 ± 0.01	2.12 ± 0.2	3.20 ± 0.1	3.4 ± 0.1
PHB content (%w/w)	43.4 ± 0.3	50.3 ± 0.3	59.5 ± 0.2	30.4 ± 0.5
Spores/ml^a^	2.6 × 10^6^	2.1 × 10^3^	0.73 × 10^2^	3.4 × 10^4^
Residual glucose (g/l)	4.8 ± 0.2	4.1 ± 0.3	3.2 ± 0.1	3.5 ± 0.3
Final pH	4.5 ± 0.01	5.1 ± 0.01	5.3 ± 0.02	4.9 ± 0.01

^a^Mean ± SD of triplicate experiments

The spores were not detected in the production media until 72 h with <80 spores/ml present at the end of 120 h. The reason for such delayed sporulation could be due to the presence of peptone as the complex nutrient which probably regulates the flux of metabolic intermediates towards PHB biosynthesis favoring growth-associated PHB production in *B. mycoides*. Earlier studies also documented on the stimulation of spore formation in response to stringent conditions (De Vries et al. 2004).

The effect of initial pH was investigated for the 3:1 *G*/*P* ratio and the results are shown in Table 2. Although cell growth and PHB production was observed from pH 5.7 onwards, the PHB content (11.3 ± 0.5 %w/w) was low at the end of 48 h. The maximum PHB accumulation (65.5 ± 1.5 %w/w) was observed at pH 7.3 with minimum sporulation and remained stable without undergoing degradation. *Bacillus* sp. strains are known to produce several metabolic intermediates such as acetate, lactate and acetoin during growth and the accumulated PHB is used as energy source at the time of sporulation (Benoit et al. 1990). In the present study, the PHB remained stable probably due to some factors suppressing the intermediates of sporulation, further confirming importance of this process parameter in the production medium.

**Table 2:** Effect of initial pH on PHB production and sporulation

Initial pH	Cdw (g/l)	PHB (%w/w)	Spores/ml^a^
5.7	0.24 ± 0.07	11.3 ± 0.5	6.3 × 10^4^
5.9	0.49 ± 0.05	16.3 ± 0.1	5.7 × 10^4^
6.1	0.66 ± 0.03	32.3 ± 0.5	5.5 × 10^3^
6.3	0.74 ± 0.03	38.3 ± 1.1	4.4 × 10^2^
6.5	0.93 ± 0.05	42.6 ± 0.1	3.1 × 10^2^
6.7	1.62 ± 0.06	52.4 ± 0.6	2.3 × 10^2^
6.9	1.86 ± 0.13	57.3 ± 0.5	1.3 × 10^2^
7.1	2.11 ± 0.07	60.2 ± 1.3	1 × 10^2^
7.3	2.23 ± 0.12	65.5 ± 1.5	0.4 × 10^2^
7.5	3.12 ± 0.16	63.7 ± 1.1	0.87 × 10^2^

Mean ± SD of triplicate experiments

^a^Average of triplicate experiments

### Characterization of PHB

The FT-IR spectroscopy of the extracted polymer showed an intense band at 1,720 cm^–1^ corresponding to the ester-carbonyl (C=O) stretching vibration (data not shown) characteristic for the short chain length monomers of PHB (Hong et al. 1999).

### Statistical optimization of PHB production

Based on the results of the preliminary experiments, CCRD was used to determine the optimized culture conditions to maximize cell growth and PHB production using GPB media. The experimental design matrix is presented in Table 3 and the data obtained were used to develop models through the second-order polynomial model equation (Eqs. 2, 3) which allows all the linear and quadratic components of the main effects and the linear-by-linear interactions to be estimated.

**Table 3:** Experimental design matrix in terms of actual, coded factors and the observed values for the responses—cell dry weight (cdw) and PHB content

Variables	Symbol	Coded levels				
		−1.68	Low (−1)	Mid (0)	High (1)	+1.68
Glucose (g/l)	*A*	2.39	7.50	15.00	22.50	27.61
Peptone (g/l)	*B*	0.80	2.50	5.00	7.50	9.20
pH	*C*	6.80	7.00	7.30	7.60	7.80

Run order	Factor A	Factor B	Factor C	Mean observed response		
				Cell dry weight (*Y*_1_) g/l	PHB content (*Y*_2_) (%w/w)	
1	15.00 (0)	5.00 (0)	7.30 (0)	4.22	70.3	
2	15.00 (0)	5.00 (0)	7.30 (0)	4.32	74.2	
3	2.39 (−1.682)	5.00 (0)	7.30 (0)	1.21	34.5	
4	22.50 (1)	2.50 (−1)	7.60 (1)	0.70	21.5	
5	7.50 (−1)	2.50 (−1)	7.60 (1)	1.45	52.7	
6	27.61 (+1.682)	5.00 (0)	7.30 (0)	1.67	33.5	
7	7.50 (−1)	2.50 (−1)	7.00 (−1)	2.24	59.4	
8	15.00 (0)	5.00 (0)	7.30 (0)	4.15	74.4	
9	22.50 (1)	2.50 (−1)	7.00 (−1)	0.82	25.3	
10	7.50 (−1)	7.50 (1)	7.00 (−1)	2.32	38.7	
11	15.00 (0)	9.20 (+1.682)	7.30 (0)	4.18	69.4	
12	15.00 (0)	5.00 (0)	7.80 (+1.682)	3.24	65.2	
13	7.50 (−1)	7.50 (1)	7.60 (1)	2.45	34.6	
14	22.50 (1)	7.50 (1)	7.00 (−1)	3.13	67.4	
15	15.00 (0)	0.80 (−1.68)	7.30 (0)	1.15	31.3	
16	22.50 (1)	7.50 (1)	7.60 (1)	3.52	61.7	
17	15.00 (0)	5.00 (0)	7.30 (0)	3.87	72.8	
18	15.00 (0)	5.00 (0)	7.30 (0)	4.17	73.3	
19	15.00 (0)	5.00 (0)	7.30 (0)	3.92	74.4	
20	15.00 (0)	5.00 (0)	6.80 (−1.682)	3.11	72.5	

23

**Fig. 3:**
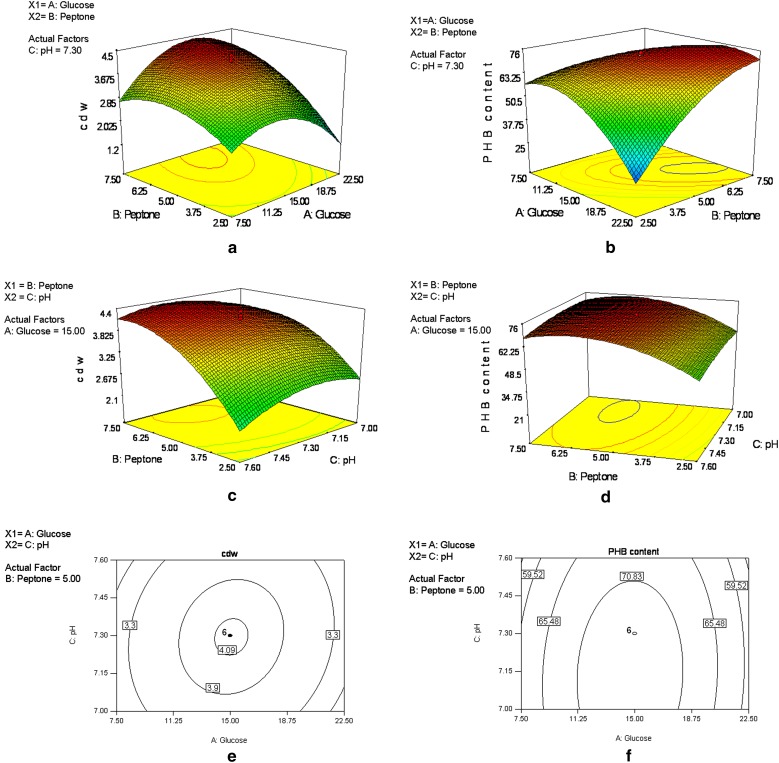
**a**–**d** Surface plots showing the interactive effect of glucose versus peptone for the responses: **a** cell dry weight (g/l) and **b** PHB content (%w/w); interactive effect shown as a function of peptone versus pH for **c** cell dry weight and **d** PHB content. **e**–**f** Interactive effect shown as a function of glucose versus pH for the responses **e** cell dry weight and **f** PHB content

The analysis of variance (Tables 4, 5) indicated that *B*, *AB*, *A*^2^, *B*^2^ and *C*^2^ as significant terms (*p* < 0.05) for cell growth and PHB content. The sign and magnitude of the coefficients indicated the effect of the variables on the responses. At quadratic level glucose (*A*) and pH (*C*) were found to have significant (*p* < 0.05) negative effect on both cell growth and PHB content and therefore can act as limiting factor at high concentrations leading to sporulation. Glucose and peptone (*B*) showed significant (*p* < 0.05) positive interactive effect for both cell growth and PHB content. In Fig. 3a–d are shown the surface plots for the interactive factors glucose, peptone and pH. The 2D contour and the 3D response surface plots are generally the graphical representation of the regression equation and the interaction between the variables can be inferred from the shapes of the surface plots (Yu et al. 2008). The maximum predicted cell dry weight (4.45 g/l) and PHB content (75.92 %w/w) increased with increase in the concentration of peptone. Further, the interaction between glucose and pH was negligible and observed as circular plots (Fig. 3e, f) when peptone concentration was kept constant at 5.0 g/l. The results indicate that the responses varied much as function of concentration of peptone and it is essential to maintain a proper *G*/*P* ratio in growth-associated PHB-producing bacteria to achieve maximum PHB production. Similar studies on the importance of the presence of complex organic nutrients favoring PHB production has been reported for some bacteria such as *Azotobacter vinelandii* (Page et al. 1992) and recombinant *E. coli* (Song et al. 1999).

**Table 4:** Analysis of regression coefficients and their significance for the response—cell dry weight (*Y*_1_)

Term	Cell dry weight (*Y*_1_)					
	Regression	SS	*df*	MS	*F* value	*p* value Prob > *F*
Intercept	4.15	31.65	9	3.52	39.57	0.0001^a^
Glucose (*A*)	0.035	0.017	1	0.017	0.19	0.6700
Peptone (*B*)	0.88	10.60	1	10.60	119.24	0.0001^a^
pH (*C*)	−0.013	2.150E−003	1	2.150E−003	0.024	0.8765
*AB*	0.51	2.05	1	2.05	23.07	0.0001^a^
*AC*	0.12	0.11	1	0.11	1.22	0.2958
*BC*	0.18	0.26	1	0.26	2.88	0.1207
*A* ^2^	−1.02	15.07	1	15.07	169.63	0.0001^a^
*B* ^2^	−0.51	3.80	1	3.80	42.78	0.0001^a^
*C* ^2^	−0.41	2.41	1	2.41	27.17	0.0004^a^
Residual	–	0.89	10	0.089		
Lack of fit	–	0.68	5	0.14	3.33	0.1602^#^
Pure error	–	0.21	5	0.041		
Total	–	32.53	19			

*SS* sum of squares, *df* degrees of freedom, *MS* mean square, *R*^2^ % = 97.2

^#^Lack of fit is not significant

^a^Significant at *p* value <0.05

**Table 5:** Analysis of regression coefficients and their significance for the response—PHB content (*Y*_2_)

Term	Regression	PHB content (*Y*_2_)				
		SS	*df*	MS	*F* value	*p* value Prob > *F*
Intercept	73.71	6,849.16	9	761.02	36.80	0.0001^a^
Glucose (*A*)	−0.82	9.16	1	9.16	0.44	0.5209
Peptone (*B*)	7.88	847.39	1	847.39	40.97	0.0001^a^
pH (*C*)	−2.39	77.71	1	77.71	3.76	0.0813
*AB*	15.14	1,833.15	1	1,833.15	88.64	0.0001^a^
*AC*	0.16	3.65	1	3.65	0.76	0.4045
*BC*	0.088	1.81	1	1.81	0.38	0.5539
*A* ^*2*^	−14.95	3,219.60	1	3,219.60	155.68	0.0001^a^
*B* ^*2*^	−9.17	1,210.83	1	1,210.83	58.55	0.0001^a^
*C* ^*2*^	−2.63	55.67	1	55.67	11.57	0.0068^a^
Residual	–	206.81	10	20.68		
Lack of fit	–	35.66	5	7.13	2.86	0.1365^#^
Pure Error	–	20.45	5	4.09		
Total	–	7,055.97	19			

*SS* sum of squares, *df* degrees of freedom, *MS* mean square, *R*^2^ % = 97.0

^#^Lack of fit is not significant

^a^Significant at *p* value <0.05

The significance and adequacy of the model was assessed using *F* test and determination coefficient (*R*^2^) of the analysis of variance (ANOVA). The model *F* value for the responses cell dry weight and PHB content were high, i.e., 39.57 and 36.80, respectively. A high model *F* value indicates the model as significant and the factors could explain adequately the variation in the data around its mean. The goodness of fit of the model determined by correlation coefficient was also high (*R*^2^ = 0.97) for both the responses, since *R*^2^ >0.75 indicates aptness of the model. The adequate precision value which measures the signal-to-noise ratio is 16.135 and 16.445 for cell growth and PHB content. A value of >4 is considered desirable and indicate that the model could navigate the design space which is further corroborated by the insignificant lack of fit values (Tables 4, 5).

### Model validation and PHB production

**Fig. 4:**
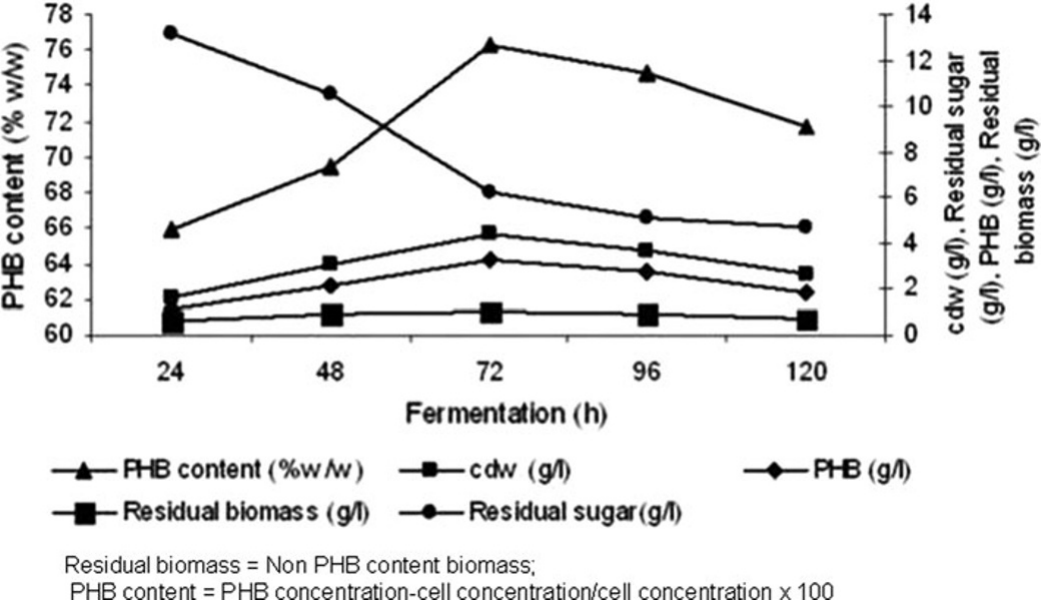
Time course study of biomass and PHB production in optimized media by *B. mycoides* DFC1

The recommended solution obtained for the constraint to ‘maximize’ cell dry weight and PHB content was glucose 17.34 g/l, peptone 7.03 g/l and pH 7.3. With the optimized solution, experiments were carried out in duplicates to evaluate the accuracy of the predicted model using the modified GPB medium. The percentages of relative deviation for the validated responses calculated for the predicted and observed response are shown in Table 6. Further the time course fermentation study (Fig. 4) revealed that the PHB content (76.32 %w/w) showed a 1.82-fold increase (1.82–3.32 g/l) after optimization using response surface methodology in comparison to the media used in the preliminary studies. The endospore staining and fluorescence microscopic studies of bacterial cells revealed negligible (~40 spores/ml) spore count for the optimized media at the end of 72 h which further substantiate the efficiency of the GPB media for PHB production. The yield obtained is comparatively higher when compared with other *Bacillus* sp. strains like *Bacillus sp* INT005 (35.30 %) (Tajima et al. 2003), *Bacillus cereus* SPV (38.0 %) (Valappil et al. 2007), *Bacillus cereus* CFR06 (46.0 %) (Halami 2008) reported so far. Although *Bacillus* sp. are considered as industrial workhorses for the production of various enzymes, antibiotics and host for recombinant DNA technology after *E. coli*, the studies on industrial scale PHB production has not drawn much attention due to the limitations of PHB as energy source for sporulation process (Singh et al. 2009; Jendrossek 2009). The present study identified a production medium with minimum ingredients for PHB biosynthesis by *B. mycoides* DFC1, which resisted sporulation for longer hours (72 h) as well as accumulated PHB to the maximum extent of ~76.0 % of its dry cell weight when compared to earlier reports.

**Table 6:** Constraints, criteria for optimization, solution along with predicted and observed response values

Name	Goal	Lower limit	Upper limit	Importance	Solution	Observed response^b^	Predicted response	Relative deviation (%)^a^
Glucose (g/l)	Is in range	7.5	22.5	3	17.34	–	–	–
Peptone (g/l)	Is in range	2.5	7.5	3	7.03	–	–	–
Initial pH	Target = 7.30	7.0	7.6	3	7.30	–	–	–
Cdw (g/l)	Maximize	0.7	4.61	4	4.20	4.35	4.50	3.33
PHB content (%w/w)	Maximize	15.7	74.4	5	75.7	76.32	75.7	−0.92

^a^

^b^Average of duplicate experiments

## Conclusion

In the present investigation, the conditions favoring maximum PHB production over sporulation was evaluated using a modified glucose–peptone medium by one-factor-at-a-time approach. The application of statistical optimization helped to determine collectively the optimum process conditions responsible for PHB production. The study also revealed that selected process parameters supported maximum PHB accumulation and also reduced the problem of sporulation. Further scale-up studies using the optimized media by *B. mycoides* DFC1 is under progress.
